# Association of polymorphisms of calcium reabsorption genes *SLC12A1*, *KCNJ1* and *SLC8A1* with colorectal adenoma

**DOI:** 10.1007/s00432-023-04773-4

**Published:** 2023-04-19

**Authors:** Xiaolian Lai, Shuoyan Lu, Jia Jiang, Hanqun Zhang, Qinglin Yang, Yuncong Liu, Libo Li, Sanming Li, Si Dai, Yanping Chen, Yan Chen, Jun Liu, Yong Li

**Affiliations:** 1 Department of Gastroenterology, People’s Hospital of Songtao Miao Autonomous County, Tongren, China; 2 Guizhou University of Traditional Chinese Medicine, Guiyang, China; 3 Department of Oncology, Guizhou Provincial People’s Hospital, Guiyang, China; 4 Department of Preventive Medicine, School of Public Health at Zunyi Medical University, Zunyi, China; 5 People’s Hospital of Fenggang County, Zunyi, China

**Keywords:** SNPs, *SLC12A1*, *KCNJ1*, *SLC8A1*, Colorectal adenomas

## Abstract

**Background:**

In recent years, morbidity and mortality from colorectal cancer have increased. Colorectal adenoma is the main precancerous lesion. Understanding the pathogenesis of colorectal adenoma will help to improve the early diagnosis rate of colorectal cancer.

**Methods:**

In this case–control study, we focused on three single nucleotide polymorphisms (SNPs) in genes *SLC8A1* (rs4952490), *KCNJ1* (rs2855798), and *SLC12A1* (rs1531916). We analyzed 207 colorectal adenoma patients (112 high-risk cases and 95 low-risk cases) and 212 control subjects by Sanger sequencing. A food frequency questionnaire (FFQ) was used to survey demographic characteristics and dietary nutrition.

**Results:**

In the overall analysis, the results suggested that the AA+AG and AG genotype carriers of rs4952490 had a 73.1% and 78% lower risk of colorectal adenoma compared to GG genotype carriers, respectively. However rs2855798 and rs1531916 were not associated with the incidence of colorectal adenoma. Additionally, stratified analysis showed that rs4952490 AA+AG and AG genotypes had a protective effect against low-risk colorectal adenoma in patients aged ≤ 60 years old who were non-smokers. We also observed that when calcium intake was higher than 616 mg/d and patients carried at least one gene with variant alleles there was a protective effect against low-risk colorectal adenoma.

**Conclusions:**

Interactions between dietary calcium intake and calcium reabsorption genes may affect the occurrence and development of colorectal adenoma.

**Supplementary Information:**

The online version contains supplementary material available at 10.1007/s00432-023-04773-4.

## Introduction

Colorectal cancer (CRC) is an increasingly common malignant tumor in the digestive system. According to global cancer burden data from the International Agency for Research on Cancer, in 2020 there were 560,000 new cases and 290,000 deaths from CRC in China (Sung et al. 2021). The American Cancer Society publishes a cancer statistics report, which estimated that by 2020, there would be 150,000 new cases of CRC in the United States and 50,000 deaths (Siegel et al. 2021). Colorectal adenoma (CRA) is the main precancerous lesion of colorectal cancer, and its evolution to colorectal cancer contains a wide range of genetic and epigenetic changes, such as chromosomal instability (CIN), micro-Satellite instability (MSI), and DNA methylation of CpG islands (Siskova et al. 2020). Colorectal cancer begins when CRA first develops in normal intestinal mucosa, and then further progresses to form adenocarcinoma (Saito et al. 2018). Exploring the mechanism of occurrence and development of CRA will help to diagnose and prevent CRC and therefore reduce morbidity and mortality from CRC.

Studies indicate that diet plays an important role in the occurrence and development of CRA. Studies on early chemoprevention of colorectal cancer reported that calcium had a potential protective role against adenoma development (Liu et al. 2020; Sharif et al. 2022; Schulz et al. 2014). Calcium absorption varies greatly by individual, and a large part of this variation is not caused by age, smoking, or dietary factors. Previous studies indicate that calcium reabsorption genes are crucial to calcium absorption (Gamba and Friedman 2009; Sharma and Linshaw 2011; Besserer et al. 2007). Recent research has found some key genes involved in calcium reabsorption and the polymorphism of these genes has a significant impact on calcium homeostasis. It was reported that polymorphisms of genes *VDR* (Slattery et al. 2004a, b; Slattery et al. 2004a, b), *CaSR* (Rogers et al. 2012; Miller et al. 2007) and *TRPM7* (Dai et al. 2007) affected calcium intake and colorectal adenoma development. Polymorphisms of genes related to calcium absorption may also play an important role. The solute carrier family 12 member 1 gene (*SLC12A1*), the potassium inward rectifier channel subfamily J member 1 gene (*KCNJ1*), and the solute carrier family 8 member 1 gene (*SLC8A1*) all are essential players in maintaining intracellular calcium homeostasis and regulating calcium transport (Li et al. 2016; Srivastava et al. 2013; Lubelwana et al. 2017). The Tennessee Colorectal Polyp Study (TCPS) reported that high calcium intake was associated with a reduced risk of colorectal adenoma in people carrying variant alleles of *KCNJ1*rs2855798, *SLC12A1*rs1531916 and *SLC8A1*rs4952490 (Zhao et al. 2017; Zhu et al. 2014). However, there has not been a study on the relationship between these three SNPs with colorectal adenoma in a Chinese population. Moreover, compared with Europe and North America, calcium deficiency is common among Chinese people. It is still unknown whether calcium deficiency modifies the relationship of three SNPs with colorectal adenoma in Chinese populations.

We conducted a case–control study in Guizou province to investigate the association of *KCNJ1*rs2855798, *SLC12A1*rs1531916 and *SLC8A1*rs4952490 with colorectal adenoma in Chinese, and explored potential gene interactions with dietary calcium.

## Materials and methods

### Study subjects

From September 2020 to September 2021, participants aged 18–75 who had a colorectal endoscopy were recruited from Guizhou Provincial People’s Hospital and the People’s Hospital of Songtao Miao Autonomous County. According to the WHO standards, the colorectal adenoma cases were diagnosed and determined by degree of neoplasia. Selection criteria was (1) Adenoma was confirmed by pathological biopsy; (2) The villous structure in villous adenoma or mixed adenoma exceeded 25%; and (3) Adenoma was accompanied by a high grade intraepithelial neoplasia. The control participants were selected from healthy participants without adenoma, who were examined during the initial screening. Patients with familial adenomatous polyposis (FAP), inflammatory bowel disease, hereditary nonpolyposis colorectal cancer (HNPCC), Turcot syndrome, severe cardiovascular and cerebrovascular diseases, adenoma recurrence, colorectal cancer or other organ tumors were excluded from the study. This study has been approved by the Ethics Committee of Guizhou Provincial People's Hospital (No. [2019]021). Written informed consent was obtained from all participants. Finally, 207 colorectal adenoma cases and 212 controls were included in the present study.

### Questionnaire

The survey was conducted in-person. Questionnaire was mainly divided into two parts: general conditions and food intake frequency. The general conditions mainly included: basic demographic characteristics, lifestyle, relevant disease history of the participants, such as age, gender, smoking, drinking, hypertension, diabetes, etc. Smokers were defined as participants who smoked at least five packs of cigarettes a year. Drinkers were defined as participants who drank alcohol at least once a week for at least six months. Adenoma patients were divided into low and high risk groups. Single tubular adenoma < 1 cm in diameter was classified as low risk. Single adenoma ≥ 1 cm in diameter, tubulovillous adenoma, villous adenoma, or patients with multiple adenoma were all classified as the high-risk group (Wieszczy et al. 2021).

A FFQ was used to investigate the dietary intake of the participants in the previous year. The FFQ included commonly consumed food groups: cereal products, vegetables, fruits, red meat, processed meat, poultry, fish, shrimp, eggs, dairy products, beans, fungi, algae, nuts, beverages and soup. Intake frequency ranged from never, annually, monthly, weekly or daily, and the weight of each food was estimated. Energy and nutrient intakes were calculated using the Chinese Food Composition Database (Yang.^2004^). The effectiveness and reproducibility of the FFQ have been previously evaluated (Zhang and Ho 2009).

### DNA isolation and Sanger sequencing

We collected 5 ml of peripheral blood samples in EDTA anticoagulant tubes and froze them at – 80 ℃. Isolated DNA was extracted from whole blood using a mammalian blood genomic DNA extraction kit (LifeFeng, China), and the purity of DNA was checked using a NanoDrop 2000 spectrophotometer (Thermo Fisher, USA). In order to find related sequences in the NCBI GenBank, we used Primer Premier 3.0 to design the primers for rs2855798, rs1531916, and rs4952490 gene sequences. Sanger sequencing was completed by Boshang Biotechnology Co., Ltd.

### Statistical analyses

Data were entered in Epidata 3.1 twice, SPSS 26.0 was used for statistical analysis, the Student’s t test was used to test differences in the continuous variables between the case and control subjects and the chi-squared (*χ*^2^) test was used to test differences in categorical variables between the case and control subjects. The odds ratio (OR) and 95% confidence interval (95% CI) were used to explore the association analysis. Age, gender, smoking status, drinking habit, hypertension history, diabetes history, calcium and magnesium intake were factors included in an unconditional logistic regression model to test for interactions, with *P* < 0.05 defined as statistically significant.

## Results

### Basic characteristics

There was no statistically significant difference in the distribution frequency of rs2855798, rs1531916, and rs4952490 genotypes between the control group and the colorectal adenoma group (*P* > 0.05). Other factors including age, gender, smoking status, hypertension and diabetes history were statistically significant between the case group and the control group (*P* < 0.05), while dietary factors including dietary calcium intake and dietary magnesium intake were not statistically significant between the two groups (*P* > 0.05) (Table 1). All SNPs followed Hardy–Weinberg equilibrium (*P* > 0.05) (Supplemental Table 1).

**Table 1:** Basic demographic characteristics

Variables	Controls, *n* (frequency)	CRA,* n* (frequency)	Statistical value	*P* value
Age ($\overline{x} \pm s$)	56.50 ± 12.83	60.86 ± 9.73	*t* = − 3.902	< 0.001
Sex (male)	124 (58.77)	142 (68.10)	*χ*^2^ = 4.364	0.037
Smoke (yes)	98 (46.45)	119 (57.49)	*χ*^2^ = 5.104	0.024
Drinking (yes)	43 (20.38)	47 (22.71)	*χ*^2^ = 0.335	0.563
Hypertension (yes)	57 (27.01)	77 (37.20)	*χ*^2^ = 4.975	0.026
Diabetes (yes)	16 (7.62)	32 (15.46)	*χ*^2^ = 6.377	0.012
Calcium intake ($\overline{x} \pm s$, mg/d)	662.97 ± 280.57	675.15 ± 232.71	*t* = − 0.483	0.629
Magnesium intake ($\overline{x} \pm s$, mg/d)	286.837 ± 109.10	296.99 ± 100.19	*t* = − 0.991	0.322
rs2855798			*χ*^2^ = 0.299	0.861
GG	152 (72.04)	149 (73.04)		
GT	52 (24.64)	50 (24.51)		
TT	7 (3.32)	5 (2.45)		
rs1531916			*χ*^2^ = 0.910	0.634
AA	47 (22.27)	38 (18.63)		
GA	102 (48.34)	104 (50.98)		
GG	62 (29.38)	62 (30.39)		
rs4952490			*χ*^2^ = 1.698	0.428
AA	136 (64.15)	138 (67.32)		
AG	71 (33.49)	59 (28.78)		
GG	5 (2.36)	8 (3.90)		

Continuous and categorical variables were described by means median or numbers and percentages, and evaluated by the Wilcoxon signed-rank or McNemar’s test, respectively, to compare the categorical and continuous variables of case and control group

### Distribution and risk assessment of SLC12A1, SLC8A1 and KCNJ1 SNP in colorectal adenoma cases and the control population

The genotypes of rs2855798, rs1531916, and rs4952490 were divided into five different genetic models. Univariate analysis found the different genetic models of rs2855798 and rs1531916 were not related to colorectal adenoma. However, the rs4950490 model had significant differences between the control group and the low-risk adenoma group. After adjusting for gender, age, smoking status, drinking habit, hypertension history, diabetes history, calcium and magnesium intake, it was found that only the recessive mode and cumulative mode of rs4952490 locus were associated with low-risk colorectal adenoma. The risk of low-risk colorectal adenoma of AA+AG genotype carriers was 73.1% lower than that of GG genotype carriers (OR = 0.269, 95% CI 0.079–0.919), and carriers of AG genotype was 0.22 times that of GG genotype (Table 2).

**Table 2:** Distribution and risk assessment of *SLC12A1*, *SLC8A1* and *KCNJ* 1 in colorectal adenoma cases and the control population

SNP ID	Genotype	Controls, *n* (frequency)	CRA(High-risk, *n* (frequency)	CRA (Low-risk),* n* (frequency)	Controls VS CRA (High-risk)		Controls *VS* CRA (Low-risk)	
					OR (95% CI)^a^	OR (95% CI)^b^	OR (95% CI)^a^	OR (95% CI)^b^
rs4952490	Codominant pattern							
	AA	136 (64.15)	79 (70.54)	59 (63.44)				
	AG	71 (33.49)	31 (27.68)	27 (29.03)	0.752 (0.454–1.452)	0.816 (0.481–1.386)	0.877 (0.512–1.502)	0.883 (0.505–1.544)
	GG	5 (2.36)	2 (1.79)	7 (7.53)	0.830 (0.361–1.906)	0.645 (0.265–1.568)	1.796 (0.992–3.253)	1.862 (0.992–3.497)
	Hyperdominant pattern							
	AG	71 (33.49)	31 (27.68)	27 (29.03)				
	AA+GG	141 (66.51)	81 (72.32)	66 (70.97)	1.316 (0.796–2.175)	1.197 (0.708–2.026)	1.231 (0.724–2.093)	1.238 (0.716–2.142)
	Dominant pattern							
	AA	136 (64.15)	79 (70.54)	59 (63.44)				
	GA+GG	76 (35.85)	33 (29.46)	34 (36.56)	0.748 (0.456–1.225)	0.788 (0.469–1.322)	1.031 (0.621–1.712)	1.040 (0.614–1.760)
	Recessive pattern							
	GG	5 (2.36)	2 (1.79)	7 (7.53)				
	AA+AG	207 (97.64)	110 (98.21)	66 (92.47)	1.329 (0.254–6.959)	1.973 (0.342–11.371)	0.297 (0.092–0.961)*	0.269 (0.079–0.919)*
	Accumulation pattern							
	GG	5 (2.36)	2 (1.79)	7 (7.53)				
	AG	71 (33.49)	31 (27.68)	27 (29.03)	1.092 (0.201–5.935)	1.827 (0.257–12.581)	0.272 (0.079–0.930)*	0.220 (0.057–0.851)*
rs2855798	Codominant pattern							
	GG	152 (72.04)	82 (73.04)	68 (73.12)				
	GT	52 (24.64)	27 (24.51)	23 (24.73)	0.962 (0.563–1.647)	0.883 (0.500–1.558)	0.989 (0.560–1.745)	0.948 (0.524–1.714)
	TT	7 (3.32)	3 (2.45)	2 (2.15)	0.825 (0.197–3.450)	0.983 (0.478–2.022)	0.639 (0.129–3.155)	0.841 (0.370–1.912)
	Hyperdominant pattern							
	GT	52 (24.64)	27 (24.51)	23 (24.73)				
	GG+TT	159 (75.36)	85 (75.49)	70 (75.27)	1.030 (0.603–1.757)	1.140 (0.647–2.008)	0.995 (0.565–1.752)	1.029 (0.571–1.855)
	Dominant pattern							
	GG	152 (72.04)	82 (73.04)	68 (73.12)				
	GT+TT	59 (27.96)	30 (26.96)	25 (26.88)	0.943 (0.563–1.578)	0.893 (0.518–1.540)	0.947 (0.547–1.639)	0.925 (0.523–1.636)
	Recessive pattern							
	TT	7 (3.32)	3 (2.45)	2 (2.15)				
	GG+GT	204 (96.68)	109 (96.55)	91 (97.85)	1.247 (0.316–4.918)	0.943 (0.228–3.892)	1.561 (0.318–7.662)	1.475 (0.289–7.534)
	Accumulation pattern							
	TT	7 (3.32)	3 (2.45)	2 (2.15)				
	GT	52 (24.64)	27 (24.51)	23 (24.73)	1.212 (0.290–5.064)	0.534 (0.110–2.605)	1.548 (0.298–8.031)	2.197 (0.344–14.019)
rs1531916	Codominant pattern							
	AA	47 (22.27)	22 (19.65)	16 (17.20)				
	GA	102 (48.34)	61 (54.46)	44 (47.31)	1.278 (0.703–2.322)	1.227 (0.657–2.290)	1.267 (0.649–2.472)	1.250 (0.628–2.490)
	GG	62 (29.39)	29 (25.89)	33 (35.48)	1.000 (0.715–1.398)	1.072 (0.738–1.559)	1.250 (0.878–1.781)	1.216 (0.837–1.767)
	Hyperdominant pattern							
	GA	102 (48.34)	61 (54.46)	44 (47.31)				
	AA+GG	107 (51.66)	51 (45.54)	49 (52.69)	0.782 (0.494–1.239)	0.825 (0.509–1.337)	1.042 (0.639–1.698)	1.079 (0.651–1.789)
	Dominant pattern							
	GG	62 (29.38)	29 (25.89)	33 (35.48)				
	GA+AA	149 (70.62)	83 (74.11)	60 (64.52)	1.191 (0.711–1.996)	1.135 (0.659–1.954)	0.757 (0.451–1.270)	0.741 (0.432–1.271)
	Recessive pattern							
	AA	47 (22.27)	22 (19.65)	16 (17.20)				
	GA+GG	164 (77.73)	90 (80.35)	77 (82.80)	1.172 (0.664–2.069)	1.149 (0.633–2.087)	1.379 (0.736–2.586)	1.340 (0.699–2.568)
	Accumulation pattern							
	GG	62 (29.39)	29 (25.89)	33 (35.48)				
	GA	102 (48.34)	61 (54.46)	44 (47.31)	1.279 (0.743–2.201)	1.218 (0.688–2.156)	0.810 (0.467–1.406)	0.777 (0.436–1.387)

*P < 0.05

^a^Uncorrected OR

^b^Adjusted for gender, age, smoking, drinking, hypertension, diabetes and calcium and magnesium intake

### Distribution and risk assessment of rs4952490 recessive pattern in colorectal adenoma cases and the control population in stratified analysis

The recessive inheritance pattern of the rs4952490 was stratified according to sex, age, smoking status, and calcium intake. Univariate analysis showed that women carrying AA+AG genotype had a protective effect against low-risk colorectal adenoma, but in multivariate analysis, no statistical correlation was found between AA+AG genotype and low-risk colorectal adenoma. Univariate and multivariate analysis both showed that AA+AG genotype carriers aged ≤ 60 years were negatively correlated with low-risk colorectal adenoma. Interaction analysis only showed that the AA+AG genotype interacted with age (*P*-interaction = 0.038), and showed that AA+AG genotype was not associated with gender, smoking status or dietary calcium intake (Table 3).

**Table 3:** Distribution and risk assessment of rs4952490 recessive pattern in colorectal adenoma cases and control population in stratified analysis

Item	Genotype	Controls, *n* (frequency)	CRA (Low-risk), *n* (frequency)	Controls *VS* CRA (Low-risk)	Controls *VS* CRA (Low-risk)	*P*-interaction
				OR (95% CI)^a^	OR (95% CI)^b^	
Sex						0.283
Male						
	GG	3 (2.42)	3 (5.00)			
	AA+AG	121 (97.58)	57 (95.00)	0.471 (0.092–2.407)	0.391 (0.073–2.102)	
Female						
	GG	2 (2.30)	4 (12.12)			
	AA+AG	85 (97.70)	29 (87.88)	0.171 (0.030–0.981)*	0.138 (0.018–1.064)	
Age						0.038*
> 60						
	GG	3 (3.30)	3 (5.77)			
	AA+AG	88 (96.70)	49 (94.23)	0.557 (0.108–2.865)	0.505 (0.090–2.825)	
≤ 60						
	GG	2 (1.67)	4 (9.76)			
	AA+AG	118 (98.33)	37 (90.24)	0.157 (0.028–0.891)*	0.102 (0.015–0.706)*	
Smoke						0.107
Yes						
	GG	2 (2.04)	1 (1.96)			
	AA+AG	96 (97.96)	50 (98.04)	1.042 (0.092–11.769)	0.744 (0.062–8.892)	
No						
	GG	3 (2.65)	6 (14.29)			
	AA+AG	110 (97.35)	36 (85.71)	0.073 (0.017–0.320)*	0.168 (0.037–0.754)*	
Calcium intake						0.112
< 616 mg/d						
	GG	3 (2.56)	3 (8.11)			
	AA+AG	114 (97.44)	34 (91.89)	0.298 (0.058–1.546)	0.229 (0.033–1.595)	
≥ 616 mg/d						
	GG	2 (2.13)	4 (7.14)			
	AA+AG	92 (97.87)	52 (92.86)	0.283 (0.050–1.596)	0.273 (0.043–1.745)	

*P < 0.05

^a^Uncorrected OR

^b^Adjusted for gender, age, smoking, drinking, hypertension, diabetes and calcium and magnesium intake

### Distribution and risk assessment of rs4952490 accumulation pattern in colorectal adenoma cases and control population in stratified analysis

The cumulative genetic model of the rs4952490 was also stratified by sex, age, smoking status, and calcium intake. Logistic regression analysis showed gender and age had a negative correlation between AG genotype carriers and low-risk colorectal adenoma risk. Women and patients ≤ 60 years old with the AG genotype had a protective effect against low-risk colorectal adenoma, however the interaction was not significant. The colorectal adenoma risk of AG genotype carriers was significantly 92.4% lower than that of GG genotype carriers in non-smokers. There was also no correlation between AG genotype and calcium intake (Table 4).

**Table 4:** Distribution and risk assessment of rs4952490 accumulation pattern in colorectal adenoma cases and control population in stratified analysis

Item	Genotype	Controls, *n *(frequency)	CRA (Low-risk), *n* (frequency)	Controls *VS* CRA (low-risk)	Controls *VS* CRA (low-risk)	*p*-interaction
				OR (95% CI)^b^	OR (95% CI)^d^	
Sex						0.328
Male						
	GG	3 (7.14)	3 (14.29)			
	AG	39 (92.86)	18 (85.71)	0.462 (0.085–2.514)	0.267 (0.040–1.786)	
Female						
	GG	2 (6.06)	4 (30.77)			
	AG	31 (93.94)	9 (69.23)	0.145 (0.023–0.926)*	0.021 (0.001–0.589)*	
Age						0.213
> 60						
	GG	3 (9.38)	3 (16.67)			
	AG	29 (90.62)	15 (83.33)	0.517 (0.093–2.881)	0.498 (0.066–3.776)	
≤ 60						
	GG	2 (4.65)	4 (25.00)			
	AG	41 (95.35)	12 (75.00)	0.146 (0.024–0.899)*	0.087 (0.009–0.794)*	
Smoke						0.033*
Yes						
	GG	2 (6.90)	1 (5.56)			
	AG	27 (93.10)	17 (94.44)	1.259 (0.106–14.977)	0.535 (0.034–8.439)	
No						
	GG	3 (6.52)	6 (37.50)			
	AG	43 (93.48)	10 (62.50)	0.116 (0.025–0.546)*	0.076 (0.012–0.502)*	
Calcium intake						0.131
< 616 mg/d						
	GG	3 (7.32)	3 (23.08)			
	AG	38 (92.68)	10 (76.92)	0.263 (0.046–1.507)	0.240 (0.036–1.614)	
≥ 616 mg/d						
	GG	2 (5.89)	4 (19.05)			
	AG	32 (94.12)	17 (80.95)	0.266 (0.044–1.601)	0.204 (0.027–1.536)	

*P < 0.05

^a^Uncorrected OR

^b^Adjusted for gender, age, smoking, drinking, hypertension, diabetes and calcium and magnesium intake

### Associations between calcium intake and colorectal adenoma risk stratified by the number of genes with variant alleles (SLC8A1, KCNJ1, and SLC12A1)

To further explore the relationship between calcium re-absorption genes, calcium intake and risk of colorectal adenoma, patients were stratified according to total calcium intake. In the overall analysis, the results suggested that the variant alleles were not associated with colorectal adenomas. However, compared with patients who did not carry variant alleles, having at least one variant allele showed a protective effect against low-risk colorectal adenoma, when calcium intake was greater than the median 616 mg/d (OR = 0.287, 95% CI 0.103–0.798). On the contrary, among patients whose calcium intake was lower than 616 mg/d, it could be suggested that the risk was increased in patients carrying variant alleles (Table 5).

**Table 5:** Associations between calcium intake and colorectal adenoma risk stratified by the number of genes with variant alleles (*SLC8A1*, *KCNJ1*, and *SLC12A1*)

Number of genes with variant alleles	Controls *VS* CRA (low-risk)		Controls VS CRA (high-risk)	
	Controls/cases	OR (95% CI)	Controls/cases	OR (95% CI)
All subjects				
0	27/18		27/15	
1	96/40	0.625 (0.310–1.260)	96/55	1.031 (0.506–2.104)
2	79/32	0.608 (0.295–1.253)	79/35	0.797 (0.378–1.682)
3	10/5	0.750 (0.220–2.561)	10/7	1.260 (0.397–3.995)
≥ 2	89/37	0.624 (0.307–1.267)	89/42	0.849 (0.409–1.763)
Calcium intake < 616 mg/d				
0	19/5		19/7	
1	50/19	1.444 (0.472–4.416)	50/31	1.683 (0.634–4.464)
2	43/12	1.060 (0.328–3.433)	43/14	0.884 (0.307–2.540)
3	5/2	1.520 (0.224–10.295)	5/2	1.086 (0.170–6.938)
≥ 2	48/14	1.108 (0.351–3.504)	48/16	0.905 (0.321–2.547)
Calcium intake > 616 mg/d				
0	8/13		8/8	
1	45/21	0.287 (0.103–0.798)*	45/24	0.533 (0.178–1.599)
2	36/20	0.342 (0.121–0.964)*	36/21	0.583 (0.191–1.784)
3	5/3	0.369 (0.069–1.982)	5/5	1.000 (0.206–4.856)
≥ 2	41/23	0.345 (0.125–0.955)*	41/26	0.634 (0.212–1.898)

*P < 0.05

## Discussion

In the present study, we explored the association between gene sequences rs4952490, rs2855798, and rs1531916 with colorectal adenoma in a Chinese population. In the overall analysis, compared with the GG genotype, carriers of the AA+AG and AG genotypes of rs4952490 showed reduced the risk of colorectal adenoma by 73.1% and 78%, respectively. Consistent with previous studies (Zhao et al. 2017; Zhu et al. 2014), we also found that *KCNJ1*, *SLC12A1*, and *SLC8A1* with variant alleles were not associated with colorectal adenoma risk in overall analysis. Related studies have explored how variations of these genes can also correlate with other diseases. In two studies in South Korea, the *SLC8A1* polymorphisms rs13017846 and rs17026156 were found to be related to Electrocardiogram (ECG) measurement (Kim et al. 2012; Hong et al. 2014). A randomized trial showed that the *KCNJ1* variation was related to hydrochlorothiazide-induced blood glucose abnormalities and new-onset diabetes (Karnes et al. 2013). Chang (Chang et al. 2011) studied genes *AQP2*rs296766 and *SLC12A1*rs12904216 and developed a clinically applicable prediction model to estimate the risk of thiazolidinedione-related edema in type 2 diabetes patients through age, gender, and genetic information.

Subsequently, the recessive inheritance mode and cumulative mode of rs4952490 were stratified by gender, age, smoking status and calcium intake and analyzed to investigate the AA+AG and AG genotypes on low-risk colorectal adenoma. The results showed that AA+AG genotype carriers had a protective effect on low-risk colorectal adenoma in people aged ≤ 60 years old, and the interaction analysis also showed a statistical correlation. Previous studies have shown the detection rate of colorectal adenoma increases with age (Yang et al. 2022; Zhou et al. 2018), however our study suggests a decreased incidence of colorectal adenoma with increasing age. The exact interaction mechanism between AA+AG genotype and age was not clearly established and may need further study.

The risk of colorectal adenoma in non-smoking patients with the rs4952490 AG genotype was 92.4% lower than non-smokers with the GG genotype. Such differences were not observed among smoking patient groups. The result was consistent with those of two previous studies (Gao et al. 2011; Ying et al. 2020). A study exploring the association between DNA repair gene SNPs and adenoma found that SNPs on the ataxia telangiectasia mutated (ATM) gene rs17503908 were negatively related to adenoma in non-smokers, compared to smokers (Gao et al. 2011). A study on a Chinese Han population showed that patients with the *APC* gene rs1804197 CA/AA genotype had a significantly higher risk of CRC among non-smokers, compared to smokers (Ying et al. 2020). In these studies, the correlation was limited in non-smokers. Smokers may not benefit from carrying this genotype because of the antagonism between SNPs and smoking exposure.

Zhao et al. (Zhao et al. 2017), suggested that the SNPs in *SLC8A1*, *KCNJ1* or *SLC12A1* alone were not related to colorectal adenoma or calcium intake, but a combination of SNP variant alleles in at least two genes could reduce colorectal adenoma when patients had a high calcium intake. The present study found that a calcium intake of 616 mg/d in patients that also carried variant alleles in at least one gene reduced colorectal adenoma risk, similar to the findings of Zhao et al. Our results were consistent with the “biosensitivity model”, that proposes that subjects with genetic susceptibility are expected to have worse results under adverse environmental conditions, or better results under favorable conditions compared to non-carriers (Belsky et al. 2009; Mitchell et al. 2011). We observed that subjects whose calcium intake was lower than the dietary reference intake level who had a variation of *KCNJ1*, *SLC12A1* and *SLC8A1* that promoted calcium consumption had high inflammation, which led to colorectal tumorigenesis. Calcium intake varies greatly by country and region. Studies have found that the daily calcium intakes of Greek, Dutch, and Danish people (Welch et al. 2009) are 1039 mg/d, 1033 mg/d, and 1011 mg/d respectively, while China (Huang et al. 2018) and India (Harinarayan and Ramalakshmi 2015) had a lower calcium intake of 369 mg/d, and 308 mg/d, respectively. A survey on dietary calcium intake of Chinese people showed calcium intake in the group aged 19–49 and the group aged > 50 was 391.3 mg/d and 376.5 mg/d respectively (Liu et al. 2012; Zhang et al. 2012). Based on these studies, calcium deficiency may be one of the reasons for the high incidence of colorectal cancer in the Chinese population.

Our study has some limitations. First, this study was a cross-sectional study and a causal relationship with CRC could not be clarified. We selected new cases within the past year to minimize a variety of factors that might affect the causal relationship. Second, collection of dietary and nutritional intake information was subject to each patient’s recall bias. We surveyed patients and repeatedly asked them to confirm their survey in order to reduce recall bias. Trained staff had experience conducting this questionnaire survey. Their consistent method reduced errors. Finally, the sample size of this study is small, which may lead to unclear stratification results. Larger sample sizes are needed to verify the interactions between calcium intake and the three SNPs rs2855798, rs1531916, and rs4950490.

## Conclusion

We studied the relationship between calcium reabsorption genes *SLC12A1*, *KCNJ1* and *SLC8A1* and colorectal adenomas. We found that *SLC8A1*rs4952490 was associated with low-risk colorectal adenoma in the Chinese study population. Furthermore, participants who carried at least one gene with variant alleles and who had a higher calcium intake were protected against colorectal adenoma. However, further large-scale studies are needed to clarify this relationship. It is hoped that dietary calcium supplements could reduce colorectal adenoma and provide some new strategies for early intervention of colorectal cancer in the future.

## Supplementary Information

Below is the link to the electronic supplementary material.

Supplementary file1 (DOCX 15 KB)

## Data Availability

The datasets used and analyzed during the current study are available from the corresponding author on reasonable request.
