# Supplementary material for: Association of polymorphisms of calcium reabsorption genes SLC12A1, KCNJ1 and SLC8A1 with colorectal adenoma
Source: J Cancer Res Clin Oncol. 2023 Apr 19;149(11):8335–44. doi: 10.1007/s00432-023-04773-4 (PMC10374786; doi:10.1007/s00432-023-04773-4)
Supplement: Supplementary file 1 — Supplementary file1 (DOCX 15 KB) [file 432_2023_4773_MOESM1_ESM.docx]

Supplemental Table 1 Test for deviation from Hardy–Weinberg equilibrium

| SNP ID | Genotype | Controls, n (frequency) | HWE p value | CRA, n (frequency ) | HWE p value |
| --- | --- | --- | --- | --- | --- |
| rs2855798 | GG | 152(72.04) | 0.337 | 149(73.04) | 0.743 |
|  | GT | 52(24.64) |  | 50(24.51) |  |
|  | TT | 7(3.32) |  | 5(2.45) |  |
| rs1531916 | AA | 47(22.27) | 0.681 | 38(18.63) | 0.628 |
|  | GA | 102(48.34) |  | 104(50.98) |  |
|  | GG | 62(29.38) |  | 62(30.39) |  |
| rs4952490 | AA | 136(64.15) | 0.224 | 138(67.32) | 0.594 |
|  | AG | 71(33.49) |  | 59(28.78) |  |
|  | GG | 5(2.36) |  | 8(3.90) |  |
